# Research Methodology to Define the Introduction of the SARS-CoV-2 B.1.429 Variant in Hawaii

**DOI:** 10.21203/rs.3.rs-378702/v1

**Published:** 2021-04-01

**Authors:** David P Maison, Vivek R Nerurkar

**Affiliations:** University of Hawaii at Manoa

**Keywords:** SARS-CoV-2, COVID-19, variant, tracking, origin, lineage, phylogenetic, Hawaii

## Abstract

Here, we present a methodology to define the origin of SARS-CoV-2 variants as exemplified by defining the introduction of the B.1.429 variant in Hawaii. We used 187 B.1.429 variant sequences from Hawai’i deposited in the GenBank and GISAID as of March 20, 2021, as an example to develop the methodology. Briefly, i) acquire sequences, ii) perform multiple sequence alignment, iii) trim the alignment, iv) remove incomplete sequences, v) remove duplicates, and vi) generate a phylogenetic tree. The tree defined the most recent common ancestor as the origin. Further, the multiple sequence alignment used to generate the phylogenetic tree identified 20 single nucleotide polymorphisms in the B.1.429 variant genome. The Centers for Disease Control and Prevention defines B.1.429 as a variant initially found in California. This variant was introduced in Hawai’i multiple times in early 2021. Based on the phylogenetic tree, we conclude that the B.1.429 variant has entered Hawai’i at different timepoints from at least seven different states in the continental United States. This information provides a tool for policy makers and public health officials in applying precision public health genomics.

## Introduction:

Precision public health genomics is a public health policy tool to track the spread of viruses.^1^ In this age of information, precision public health genomics has been used as a public policy tool during the West Africa Ebola Outbreak from 2014–2016.^1^ During the current Coronavirus Disease 2019 (COVID-19) pandemic, for precision public health genomics to be effective, it requires high-speed and low-cost data analysis. Since January 2020, over 1 million SARS-CoV-2 whole-genome sequences have been deposited in the GenBank and global initiative on sharing all influenza data (GISAID). A fast, effective, consistent, and economical method is required to analyze the vast amount of SARS-CoV-2 sequences and to determine the origin of SARS-CoV-2 variants and lineages in populations.

Several researchers do not have access to the supercomputing infrastructure necessary to conduct the phylogenetic analysis to determine the introduction of SARS-CoV-2 in a population and assign its origin. A simplified method is required to define the origin of SARS-CoV-2 lineages and variants using a personal computer. Using B.1.429 variant as an example, initially found in California and later identified in Hawai’i,^2^ we demonstrate a method to define the origin of SARS-CoV-2 lineages and variants using a personal computer. This method uses a combination of free online services and purchasable software. The nomenclature system developed by Rambaut and colleagues^3^ for SARS-CoV-2 lineages to assist genomic epidemiology has allowed for manageable and efficient partitioning of sequences for rapid determination of SARS-CoV-2 origin. This method partitions sequences by Pangolin Lineages into manageable multiple sequence alignments (MSA) to perform phylogenetic analysis.^3^

## Methods:

Here, we present a method to define SARS-CoV-2 variants’ origin. We used 187 B.1.429 variant sequences from Hawai’i deposited as of March 20, 2021, in the GenBank and GISAID as an example. In brief, the method includes: 1) The lineage-defining sequences of SARS-CoV-2 Lineage A and Lineage B are used as ancestral roots. ^3^ Lineage A (EPI_ISL_406801) is acquired from GISAID and Lineage B (MN908947) is acquired from GenBank. 2) To identify lineages of interest in an area, filter GISAID by location (e.g.: North America/USA/Hawaii) and download all sequences. GISAID sequences were downloaded in batches due to GISAID maximum download size. Similarly, all geographically similar sequences reported in GenBank were downloaded using the search term SARS-CoV-2 and state abbreviation (e.g.: “SARS-CoV-2 HI”) and the filter sequence length (20,000–40,000). 3) Combine the GISAID and GenBank sequences into one .fasta file and assign lineages using Pangolin Lineage Assigner (pangolin.cog-uk.io). ^3–5^ 4) Download the results to Microsoft Excel, using advanced filter to copy unique records of lineages to a new column (ex: column M), then use COUNTIF (e.g.: =COUNTIF($B$2:$B$1392,M2)) to determine prevalence of each lineage. 5) Identify sequences that are the lineage of interest (e.g.: B.1.429), from using Pangolin Lineage Assigner, and download those sequences from GenBank. Filter GISAID by the lineage of interest (e.g.: B.1.429) and download all sequences. 6) Combine lineage of interest (B.1.429) GenBank sequences, GISAID (B.1.429) sequences, EPI_ISL_406801, and MN908947 into one fasta file. 7) Align sequences using multiple alignment using fast Fourier transform (MAFFT) server (https://mafft.cbrc.jp/alignment/server/add_fragments.html?frommanualnov6)^6–8^ with MN908947 as a reference and do not remove any uninformative sequences and all parameters set as “same as input.” 8) Remove the newly added MN908947 sequence that MAFFT places at the beginning of the alignment, if not MN908947 sequence will be removed by the sRNAtoolbox in a later step and Lineage B will not be represented as an ancestral root in the phylogenetic tree. 9) Import MSA file into Geneious Prime 2021.0.3 (http://www.geneious.com), search for the orf1a 5’ start of the entire alignment (5’-atggagagccttgtccctggtttca-3’) and remove the 5’ untranslated region (UTR) by deleting the upstream region (~ 265 bp) from the MSA. Next, search for ORF10 3’ end (5’-tgtagttaactttaatctcacatag-3’) and remove the entire 3’ UTR by deleting the downstream region (~ 229 bp) from the MSA. 10) Create a duplicate file for the MN908947 sequence and remove the 5’ UTR and 3’ UTR from MN908947 based upon the aforementioned MSA. 11) Using MAFFT, load the trimmed MSA with the trimmed MN908947 as a reference and remove uninformative single sequences, e.g. uncalled nucleotides ‘n.’ Set the parameter in the MAFFT at > 0%. 12) Again, remove the additional MN908947 sequence that MAFFT places at the beginning of the alignment. 13) Using sRNAtoolbox ( https://arn.ugr.es/srnatoolbox/helper/removedup/),^9^ load the updated alignment to remove duplicate sequences and merge identifications (ID) (accession) of duplicates. This merger will create “appendages” in the phylogenetic tree where identical sequences will be lined up together with equal signs (=). 14) Import the final alignment into Geneious Prime and create approximately maximum-likelihood phylogenetic tree using FastTree program. ^10^

Additionally, assign Lineage B as the reference sequence, and use the Geneious Prime “Find Variations/SNPs” Annotate and Predict function to identify consensus SNPs. Input SNPs into the SnapGene (Insightful Science, snapgene.com) to identify the nucleotide and amino acid number and substitution as described previously.^11^

## Results:

From the 1,390 total sequences deposited in the GenBank and GISAID from Hawai’i, 41 lineages were identified with Pangolin Lineage Assigner using the advanced filter for unique records and COUNTIF function in Microsoft Excel as described in the methods section. Based on this analysis, the B.1.429 variant is 13.5% (187) prevalent among all lineages in Hawai’i.

In the GenBank, 12 of 82 Hawai’i sequences were of lineage B.1.429 as determined with the Pangolin Lineage Assigner. Total B.1.429 lineages reported worldwide in GISAID are 10,353 as determined by applying the GISAID lineage filter. Of the 10,367 sequences, 7,847 sequences were removed for being uninformative and containing incomplete sequences as described in the methods section. Further, the sRNAtoolbox server removed 574 sequences containing duplicate sequences and 15 sequences of duplicate ID. The final alignment of 1,931 sequences defined the origin of the B.1.429 variant introduced into Hawai’i.

**Figure 1:**
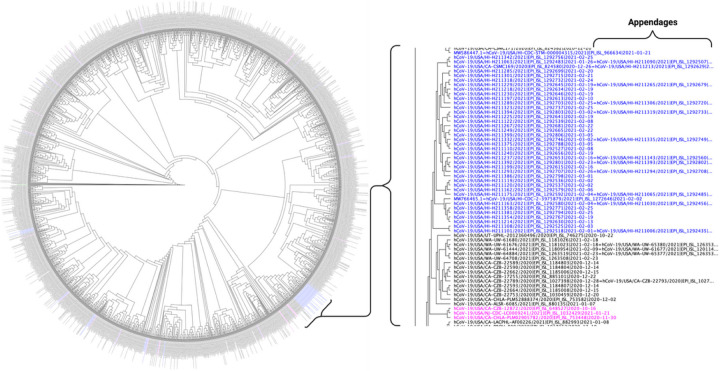
Phylogenetic Tree of B.1.429 Variants Sequenced Worldwide. This figure shows an approximately maximum-likelihood tree generated by FastTree in Geneious Prime 2021.0.3 (http://www.geneious.com). The tree was rooted to the SARS-CoV-2 Lineage A reference sequence (EPI_ISL_406801). Blue text indicates sequences from Hawai’i. The pink text indicates the most recent common ancestor sequence(s) of B.1.429 in Hawai’i. Clusters and sequences identified with the colored text (blue) were evaluated for the most recent common ancestor to define the origin of the variant sequence (pink). Text shown in black indicates global B.1.429 variant sequences not necessarily directly affiliated with variants found in Hawai’i. Appendage sequences designated by an equal sign (=) indicate sequence identifications in which the sequences were identical as generated by sRNAtoolbox. Created with BioRender.com.

**Figure 2:**
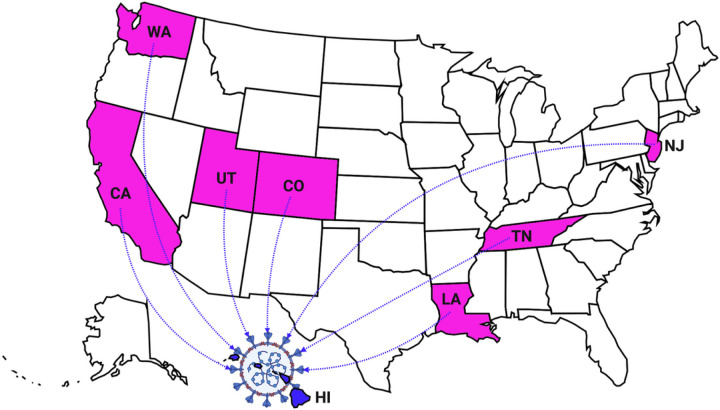
Introduction of the SAR-CoV-2 B.1.429 Variant in Hawai’i from the Continental United States. This figure shows the states within the United States from which the SARS-CoV-2 B.1.429 variant has been introduced into Hawai’i. Represented States are Tennessee, California, Utah, New Jersey, Louisiana, Colorado, and Washington (shown in pink). Figure made with a stock image by Clker-Free-Vector-Images from Pixabay. Stock image editing done in Adobe Photoshop 21.2.0. Created with Biorender.com

Figure 1, shows the phylogenetic relationship among all, 1,931, B.1.429 sequences reported worldwide rooted with the Lineage A and Lineage B reference sequences.^3^ This tree is generated using FastTree in Geneious Prime and rooted with EPI_ISL_406801.^10^ Figure 2, shows the states in the continental United States that were identified as being the source of the B.1.429 variant introductions into Hawai’i. The consensus of the B.1.429 MSA of the 1,931 sequences revealed 20 single nucleotide polymorphisms (SNP) (Table 1) as compared to the MN908947 Lineage B reference sequence.

## Discussion:

The most recent common ancestor (MRCA) branch to the sequences from Hawai’i indicates that the B.1.429 variant was introduced into Hawai’i independently and at different times from several different locations from continental United States (California, Colorado, Louisiana, New Jersey, Tennessee, Utah, and Washington). The largest cluster of 40 SARS-CoV-2 B.1.429 variants in Hawai’i, with samples collected between January and early March 2021, originated from a SARS-CoV-2 strain (EPI_ISL_753448) collected in California on November 30, 2020, at the Children’s Hospital Los Angeles. That said, the first identified, ancestral, unambiguous, and unique B.1.429 variant in Hawai’i originated from a SARS-CoV-2 strain (EPI_ISL_855068) from a sample collected on January 06, 2021, in San Juan Capistrano, California.

Within the S gene of the B.1.429 variant, there is a consensus of four non-synonymous amino acid substitutions (S13I, W152C, L452R, D614G). The effects of S13I and W152C substitutions have yet to be determined. Neither of these mutations were found in more than 5% of all published GISAID sequences as of February 2021. The L452R substitution originating in North America did so in Los Angeles (EPI_ISL_1303471), and is considered a substitution of two variants of concern (B.1.427 and B.1.429).^12^ The CDC notes that these variants correlate to ~ 20% increased transmissibility of SARS-CoV-2,^13^ and has reduced neutralization using convalescent sera, vaccinated sera, and sera from patients treated with therapeutics.^12^ The D614G substitution, near ubiquitous among all SARS-CoV-2 sequences, is noted for increasing the fitness of SARS-CoV-2.^14^

This method demonstrates the critical importance of high quality sequencing and the need for enrichment and deep coverage, as over 76% of B.1.429 published sequences worldwide are uninformative phylogenetically due to incompleteness. For example, the first B.1.429 sequence deposited from Hawaii was from a sample collected on December 31, 2020 (EPI_ISL_967766). Without resequencing the whole genome, or targeting this ambiguous region with Sanger sequencing, this sequence is currently unable to be used in phylogenetics due to ambiguous nucleotides in the S gene. While uninformative sequences may be useful for tracking specific mutations, they are not useful in tracking variants. Moreover, our methodology demonstrates the ability of sequencing and phylogenetic analysis to provide precision public health genomics in policy-making decisions. That is, as SARS-CoV-2 variants spread asymptomatically across the United States, it is important to use methods for fast and accurate SARS-CoV-2 variant, lineage, and origin assignment.

## Limitations:

FastTree is not the most stringent method for phylogenetic analysis as the bootstrapping method is not applied,^15^ even though it is one of the fastest in generating an approximately maximum-likelihood tree.^16^ FastTree is suited for large-scale phylogeny estimation, a critical component of this pandemic.^16^ Other phylogenetic softwares require substantial computational infrastructure and time to perform bootstrap analysis. Given the real-time introduction and spread of variants, a program capable of generating trees in a short amount of time without personal access to a supercomputer is warranted. Additionally, FastTree allows appendages to exist, linking identical sequences’ accession identifiers - a necessary step for the number of available SARS-CoV-2 sequences and the sequence influx.

## Figures and Tables

**Table 1.** B.1.429 Consensus (>90%) Single Nucleotide Polymorphisms and Amino Acid Substitutions as Determined From 1,931 Unique and Unambiguous B.1.429 Lineage Sequences

	B.1.429			
Gene or region	Nucleotide Loci	Nucleotide Change	Amino Acid Position	Amino Acid Change
**orf1ab**	1,059	C → T	265	Thr → Ile
	2,395	C → T	710	Val → Val
	2,597	T → C	778	Leu → Leu
	3,037	C → T	924	Phe → Phe
	8,947	C → T	2,894	Asn → Asn
	12,100	C → T	3,945	Ala → Ala
	12,878	A → G	4,205	Ile → Val
	14,408	C → T	4,715	Pro → Leu
	17,014	G → T	5,584	Asp → Tyr
**S**	21,600	G → T	13	Ser → Ile
	22,018	G → T	152	Trp → Cys
	22,917	T → G	452	Leu → Arg
	23,403	A → G	614	Asp → Gly
	24,349	T → C	929	Ser → Ser
**ORF3a**	25,563	G → T	57	Gln → His
**M**	26,681	C → T	53	Phe → Phe
**ORF7a/ORF8 intron**	27,890	G → T	-	-
**ORF8/N intron**	28,272	A → T	-	-
**N**	28,887	C → T	205	Thr → Ile
	29,362	C → T	363	Phe → Phe
